# Physical Exercise Counteracts Impaired Cognition by Improving Mitochondrial Function

**DOI:** 10.3390/ijms27104337

**Published:** 2026-05-13

**Authors:** Pedro Maciel, Caroline Barbalho Lamas, Adriano Cressoni Araújo, Eduardo F. B. Chagas, Elen Landgraf Guiguer, Rui Curi, Tania Cristina Pithon-Curi, Mariana Cristina da Silva Almeida, Kátia C. Portero Sloan, Lance A. Sloan, Ana Luiza Decanini Miranda de Souza, Claudio J. Rubira, Claudemir G. Mendes, Márcia Gabaldi Rocha, Vitor E. Valenti, Sandra M. Barbalho

**Affiliations:** 1 Department of Biochemistry and Pharmacology, School of Medicine, University of Marília (UNIMAR), Avenida Higino Muzzi Filho, 1001, Marília 17525-902, São Paulo, Brazil; 2 Department of Gerontology, School of Gerontology, Universidade Federal de Sao Carlos (UFSCar), Sao Carlos 13565-905, São Paulo, Brazil; 3 Postgraduate Program in Structural and Functional Interactions in Rehabilitation, University of Marília (UNIMAR), Marília 17525-902, São Paulo, Brazil; 4 Department of Biochemistry and Nutrition, School of Food and Technology of Marília (FATEC), Marília 17500-000, São Paulo, Brazil; 5 Butantan Institute, São Paulo 05503-900, São Paulo, Brazil; 6 Department of Cardiovascular and Metabolic Health, School of Philosophy and Sciences, Universidade Estadual Paulista (UNESP), Marilia 17525-900, São Paulo, Brazil; 7 Texas Institute for Kidney and Endocrine Disorders, Lufkin, TX 75904, USA; 8 Department of Endocrinology, University of Texas Medical Branch, Galveston, TX 77550, USA; 9 Systematic Reviews Center for Cardiovascular and Metabolic Health, School of Philosophy and Sciences, São Paulo State University, Marília 17525-900, São Paulo, Brazil; 10 UNIMAR Charity Hospital, Universidade de Marília (UNIMAR), Marília 17525-902, São Paulo, Brazil

**Keywords:** mitochondrial dysfunction, cognitive impairment, physical activity, oxidative stress, inflammation

## Abstract

Mitochondrial dysfunction is a key contributor to cognitive impairment, directly affecting neuronal viability, synaptic function, and energy metabolism. In the central nervous system, where energy demand is particularly high, disturbances in mitochondrial dynamics, including impaired oxidative phosphorylation (OxPhos), increased reactive oxygen species (ROS) production, and reduced ATP availability, can compromise synaptic transmission and accelerate cognitive decline. These alterations are commonly observed in neurodegenerative diseases such as Alzheimer’s (AD) and Parkinson’s (PD), in which mitochondrial dysfunction is closely associated with oxidative stress and neuroinflammatory processes. This review aims to investigate the role of mitochondrial dysfunction in cognitive impairment and the effects of physical exercise as a non-pharmacological strategy to mitigate these alterations. Current evidence indicates that exercise promotes mitochondrial biogenesis through activation of the AMPK/PGC-1α pathway, enhances oxidative metabolism, and improves mitochondrial efficiency. Furthermore, exercise reduces oxidative stress and inflammation while stimulating the release of neurotrophic factors, such as brain-derived neurotrophic factor which support neurogenesis, synaptic plasticity, and neuronal survival. Overall, these findings reinforce the importance of mitochondrial integrity in maintaining cognitive function and highlight physical exercise as a promising strategy to counteract mitochondrial dysfunction and delay the progression of neurodegenerative diseases.

## 1. Introduction

Mitochondrial dysfunction encompass a heterogeneous group of deficits that interfere with cellular energy metabolism [1,2,3,4,5] and are directly associated with tissues that have the highest energy demands. In the central nervous system (CNS), the brain is a significant consumer, accounting for approximately 20% of the body’s oxygen during activities, including neurotransmitter synthesis by neurons and synapses [6,7,8,9,10]. Thus, it is expected that the cognitive impairment phenotype associated with mitochondrial disorders is recurrent, with interference with neuronal activity being a step towards cognitive decline [11,12,13,14]. However, studies show that physical activity, with additional benefits from resistance and aerobic exercise, helps mitigate cognitive impairment triggered by mitochondrial dysfunction. Furthermore, research has shown that exercise can prevent approximately 3% of all existing dementia cases [15,16,17,18].

Regular exercise plays a significant role in stimulating mitochondrial biogenesis by activating AMP-activated protein kinase (AMPK), which, in turn, stimulates peroxisome proliferator-activated receptor gamma coactivator 1-alpha (PGC-1α), a key regulator of mitochondrial biogenesis [19,20]. Conversely, the oxidative phosphorylation (OxPhos) protein complexes NADH: ubiquinone oxidoreductase and cytochrome c oxidase undergo adaptations that reflect increased mitochondrial efficiency [21,22,23,24,25]. Other evidence suggests that increased glucose uptake is associated with greater availability of glucose transporters, resulting from increased cellular energy demand. This mechanism contributes to improved insulin sensitivity in peripheral tissues, in addition to reducing oxidative stress and levels of inflammatory markers such as interleukin-6 (IL-6) and tumor necrosis factor alpha (TNF-α) [26,27,28,29,30,31,32,33,34,35].

Furthermore, exercise stimulates brain neurogenesis by releasing brain-derived neurotrophic factor (BDNF), a myokine released during muscle contraction that crosses the blood–brain barrier and acts on brain plasticity, cognitive function, and neuronal survival [36,37,38]. These and other factors related to physical activity improve mitochondrial function and combat the onset and progression of neurodegenerative diseases. These diseases reflect different pathophysiological conditions in which neuronal death occurs gradually [39]. Aging is the leading risk factor for neurodegenerative diseases, which exacerbates the socioeconomic impact in countries with high life expectancy.

Currently, it is estimated that approximately 6.9 million Americans aged 65 and older live with AD. This number could double to 13.8 million by 2060, hindering the advancement of prevention or cure techniques [40]. In the case of dementia, which causes brain dysfunction due to structural and functional changes in the brain’s vascularization, the global incidence has already exceeded 50 million cases, with physical inactivity being one of the factors that influence the severity of the disease [41,42]. In this regard, evidence suggests that maintaining cerebrovascular integrity through physical training can delay or prevent dementia [41,43].

Another factor linked to these conditions is the accumulation of misfolded proteins in the brain. The deposition of misfolded proteins inside or outside nerve cells can disrupt the production and transport of molecules, disrupt synapses, and alter neuronal conformation, factors that are not yet fully understood [44,45]. Mitochondrial dysfunction is present in most neuropathies, such as AD, the most prevalent disease characterized by gradual cognitive and behavioral decline, in which levels of beta-amyloid oligomers (AβOs) and tau protein tangles are elevated, leading to neurotoxicity. Moreover, PD, which involves the loss of dopaminergic neurons in the substantia nigra, is characterized by impairment of multiple functions due to alterations in the expression of many genes [46].

HD is an autosomal dominant and lethal disorder that causes neuronal degeneration in the striatum with progressive involvement of the cerebral cortex and thalamus [47]. Friedreich’s ataxia (FA) [48,49] and amyotrophic lateral sclerosis (ALS) are characterized by motor neuron involvement in the spinal cord, cortex, and brainstem [50,51,52].

Multiple sclerosis is characterized by demyelination of the myelin sheath that surrounds neurons, resulting from an autoimmune response that leads to inflammation and potential axonal damage, thereby interrupting nerve conduction [26,53]. Finally, these and other neurological diseases that may be associated with mitochondrial imbalance highlight the signs and symptoms of dementia, a factor that can be delayed or avoided by patients who adopt a lifestyle including physical exercises [54,55,56].

Given the above, this article examines how mitochondrial dysfunction contributes to cognitive impairment and how exercise can help preserve brain function by enhancing mitochondrial health.

## 2. The Role of Mitochondria in Brain Health

Among the tissues and organs of the human body, the brain is one of the primary consumers of energy, derived from mitochondrial processes [1,2,3,4]; brain activity is a continuous process of energy expenditure in the form of ATP. This process occurs preferentially via glucose catabolism, which is imported into cells via glucose transporters. The key glucose transporters (GLUTs) include GLUT1, located at the blood–brain barrier (BBB), and GLUT3, which is predominant in neurons; both are crucial for glucose entry. Other GLUTs, such as GLUT2 in astrocytes, aid in glucose sensing, while GLUT5 is found in microglia, and GLUT4 is insulin-regulated and present in various brain regions [5,6,7]. This process consists of two main pathways: glycolysis and OxPhos [1,8].

The pathways are even used to sustain the brain’s resting state, which does not mean zero activity in the organ. This state, also known as the basal state, refers to the brain’s intrinsic activities. In contrast, the active state requires greater energy expenditure for stimulus processing and task performance [9,10].

In this sense, when addressing the energy issue, it is essential to portray neurons as polarized cells with a cell body, dendritic branches, and an axon that is primarily elongated in peripheral nerves. Even so, these components can vary and require substantial energy to maintain biological activity [11,12]. Furthermore, within this cell type’s classifications, excitatory neurons are known to consume up to three times as much energy as inhibitory neurons [13,14].

There are hypotheses that the metabolic and molecular profiles of glial cells and neurons are distinct, with glycolysis being the most relevant pathway for astrocytes. In contrast, OxPhos is more relevant in neurons [15,16,17] in addition to astrocytes, other glial cells such as oligodendrocytes and microglia, are not directly affected by mitochondrial disorders in the same way as neurons because they primarily rely on glycolysis as their primary energy pathway and have been shown to possess greater antioxidant capacity than neurons [18]. However, new studies are still investigating this hypothesis.

From this perspective, after astrocytes capture glutamate in the synaptic cleft, glucose entry into these cells is stimulated. As a result, glucose is generally not fully oxidized during glycolysis, resulting in the production of lactate as the primary product, which is subsequently converted into pyruvate, which fuels the respiratory chain in neurons [19,20,21,22].

Based on the above, it is crucial to understand how energy is utilized in subcellular brain processes, such as action potential propagation, neurotransmitter and synaptic vesicle (SV) recycling, calcium fluxes (Ca^2+^) in presynaptic terminals, postsynaptic currents, and cellular maintenance and cleanup mechanisms. Therefore, gray matter in the brain consumes approximately three times as much energy as white matter, while synaptic density is 80 times greater. Synaptic transmission is the brain’s main energy-intensive activity, involving calcium fluxes into presynaptic vesicles, the reuse of neurotransmitters in the synaptic cleft, and postsynaptic currents [23]. In white matter, the processes of homeostasis and maintenance of resting potential predominate energetically [7,24].

These processes, when compared with the action potential and synaptic transmission that occur in the same location, demonstrate greater energy expenditure, resulting in a region of higher synaptic density [24,25]. Several reactions that occur at presynaptic terminals are ATP-dependent, including synaptic vesicle reuse, endocytosis, exocytosis (priming), and vesicle acidification via H^+^-ATPases. Furthermore, voltage-gated sodium–potassium channels (Na^+^/K^+^ channels) and plasma membrane Ca^2+^-ATPase (PMCA) are ion channels present in the presynaptic membrane that utilize ATP for their activity, particularly the latter, which removes calcium accumulated in the intracellular space and transports it to the extracellular environment of neurons [26].

In active synapses, although the proportion of ATP required for each presynaptic process is not fully understood, both glycolysis and OxPhos are necessary. During nerve stimulation, for example, mitochondria become voluminous, and the density of mitochondrial cristae increases, a factor indicating increased respiratory chain activity [27]. Additionally, protein synthesis is increased upon stimulation, as is the recruitment of axonal mitochondria to presynaptic terminals. It is also understood that mitochondrial translation disruption is detrimental to synapses, underscoring the importance of protein synthesis in this organelle [28].

## 3. Mitochondrial Dysfunction in Cognitive Decline

Mitochondria are cellular organelles enclosed by two membranes. They are abundant in eukaryotes [29], having a crucial role in the production of adenosine triphosphate (ATP) in the stages of cellular respiration, with OxPhos being the phase with the highest energy balance, where the flow of electrons through protein complexes associated with the inner membrane results in the pumping of protons to the intermembrane space and subsequently to the production of energy through the enzyme F1F0-ATP synthase that converts ADP and Pi into ATP. Other functions, such as calcium storage and subcellular signaling, are also mediated by this organelle and are crucial for neuronal activity [30,31,32]. Furthermore, the organelle is involved in the production of amino acids and steroids, as well as in regulating cell death [33]. Mitochondrial impairment is generally characterized by reduction in cellular respiration parameters. This includes changes in the rate of oxygen consumption during respiration stimulated by substrates and adenosine diphosphate (ADP) (state 3), in basal respiration with only substrates (state 4), or in the respiratory control ratio (RCR).

Other indicators include decreased activity of specific enzymes, mutations or losses in mitochondrial DNA (mtDNA) [34], and increased markers of oxidative stress, such as protein, lipid, and nucleic acid oxidation [35,36,37,38,39]. This impairment may be linked to different origins than those in which the organism ages and presents a reduction in the volume of mitochondrial DNA (mtDNA), also associated with hereditary mechanisms that evidence the pathogenic mutation of mtDNA or nuclear DNA (nDNA), as well as by gene expression or mtDNA transcription errors, which distort the coding of proteins that act in mitochondrial respiration [40,41,42,43]. When it comes to aging, dysfunction is closely linked, as evidenced by impaired mitochondrial dynamics, reduced mitophagy, and the accumulation of mitochondrial DNA mutations. These processes are interconnected, such that aging marks the interplay among mitochondrial dysfunction, oxidative stress, and inflammatory processes [44].

Although the relationship between cognitive decline and mitochondrial dysfunction is strongly linked to advancing age, the association between these factors has numerous causes that have not yet been fully elucidated. Primary mitochondrial disease, classified by genetic mutations in nDNA or mtDNA, is one of the most common forms of functional organelle impairment, affecting approximately 0.02% of the world population and potentially involving maternal inheritance [45].

Proteins encoded by the nuclear genome regulate the expression and maintenance of mtDNA. Therefore, mutations in nDNA indirectly imply instability in the mitochondrial genome, leading to mitochondrial DNA depletion or deletions, which in turn cause mitochondrial disease [45,46].

In addition, nDNA can act in compensatory cellular stress pathways when mitochondrial DNA is dysfunctional, such as in mitophagy and the antioxidant system. However, mitochondrial function may be impaired in the long term due to incompatibility between the nuclear and mitochondrial genomes and disruption of mitochondrial gene expression [45]. In the context of neurodegenerative diseases, impaired mitochondrial morphology characterizes their functional impairment and the progression of cognitive decline.

It has been observed that in both AD and HD, the expression levels of OPA1 Mitochondrial Dynamin Like GTPase (OPA1), Mitofusin-1 (MFN1), and Mitofusin-2 (MFN2) expression levels are reduced, while Mitochondrial fission 1 protein (FIS1) and Dynamin-Related Protein (Drp1) are elevated, which characterizes organelle fragmentation by modifying processes such as mitochondrial fission and fusion [47]. Similar to these diseases, mitochondrial fission–fusion imbalance also occurs in PD, where pathogenic mutations in Leucine-rich repeat kinase 2 (LRRK2) increase LRRK2 kinase activity, leading to increased Drp1 and reduced OPA1 [48].

The increased activity of this enzyme is related to impaired mitophagy in these patients [48]. In Friedreich’s ataxia, mitochondrial dysfunction results from a deficiency of the frataxin protein, caused by pathogenic mutations in the FXN gene [49]. This protein is involved in iron homeostasis, iron–sulfur cluster assembly, heme biosynthesis, and intracellular redox reactions. This deficiency leads to mitochondrial iron accumulation, which generates free radicals, induces oxidative stress, and causes cell death [49]. These and other mechanisms related to cognitive decline will be discussed later in this review. It is also known that neurological disorders, such as HD, PD, and AD, have been linked to a decrease in ATP production, primarily due to dysfunctional mitochondria [50]. However, it remains unclear exactly how this occurs, as many fundamental aspects of synaptic metabolism remain poorly understood, including how ATP dysregulation affects synaptic function [51]. Therefore, it is essential to examine evidence related to the energy issues associated with synapses in neurodegenerative diseases. In AD, for example, the presence of dysfunctional mitochondria is the primary cause of synapse impairment, as ATP depletion affects neurotransmitter release, synaptic vesicle recycling, and the maintenance of ionic gradients, such as Ca^2+^-ATPases [52].

**Figure 1:**
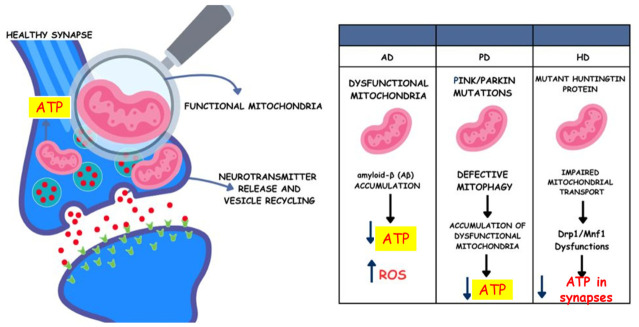
Under healthy conditions, the mitochondria present in the synaptic region are functional and produce the necessary amount of energy for nerve transmission, as well as the release of neurotransmitters and recycling through vesicles without failure. Mitochondrial dysfunction has distinct effects on different neurodegenerative diseases. In AD, the accumulation of β-amyloid increases ROS production and impairs mitochondrial transport to energy-demanding sites, leading to decreased local ATP. In PD, mutations in the PINK1 and Parkin proteins directly impair mitophagy, leading to the accumulation of damaged mitochondria and reduced ATP production. Finally, in HD, the proteins Drp1 and Mfn1, responsible for mitochondrial fission and fusion, respectively, are dysfunctional. In addition, mutations in the huntingtin protein impair mitochondrial transport to synapses, leading to energy deficits in these regions. Abbreviations: ATP; Adenosine Triphosphate; ROS, Reactive Oxygen Species; PINK, PTEN-induced kinase; Parkin, Parkin RBR E3 ubiquitin protein ligase; Drp1, Dynamin-related protein 1; Mfn1, Mitofusin 1.

Furthermore, synaptic mitochondria are potentially affected by amyloid-β (Aβ) accumulation in the brain in AD, which decreases mitochondrial transport to energy-dependent synaptic sites, compromises respiratory function, and increases ROS production, thereby affecting nerve transmission. In PD, the ATP demand of dopaminergic neurons in the substantia nigra is significantly elevated, leading to oxidative stress and consequent mitochondrial depolarization or uncoupling. The damaged mitochondria must then be removed or replaced due to the low energy supply to synapses in this region. However, the proteins involved in mitophagy (PINK1 and Parkin) are often mutated in this disease, highlighting insufficient energy for neurotransmission. In HD, the mutated huntingtin protein (mhtt) directly interferes with mitochondrial trafficking to synapses even before the onset of symptoms and loss of nerve transmission, resulting in the accumulation of fragmented mitochondria in the cell body due to dysfunction of the proteins Drp1 (responsible for mitochondrial fission) and Mfn1 (responsible for mitochondrial fusion). Impaired trafficking leads to ATP shortages at synapses [23]. Finally, it is worth highlighting the need for a better understanding of the future of energy supply at synapses (Figure 1).

## 4. Free Radicals Production, ROS Balance, and Oxidative Stress

Reactive oxygen species (ROS) are a class of molecules with high reactivity compared to molecular oxygen. They are composed of at least one atom of this chemical element, which has a high oxidizing potential and possesses two unpaired electrons in the valence shell. This class encompasses a variety of chemical species beyond the radicals produced, including hydrogen peroxide (H_2_O_2_), superoxide (O_2_^−^), peroxide (O_2_^2−^), hydroxyl radical (OH), and hydroxyl ion (OH^−^) [53,54]. Among these species, the hydroxyl radical is the most reactive and, in excess, can cause cytotoxic effects [55].

Cellular ROS are typically generated from both exogenous and endogenous sources. External sources include ultraviolet (UV) radiation, ionizing radiation, drugs that trigger ROS production, and toxins. In endogenous processes, the mitochondrial respiratory chain is the primary source of reactive species, whose production is modulated primarily by its protein complexes. Superoxide (O_2_^−^) is formed primarily by complexes I, II, and III, with NADH dehydrogenase producing the most O_2_^−^ in the brain and participates in the transfer of electrons from NADH to the third complex. Succinate dehydrogenase is recognized for its low contribution to the production of this type of ROS, being more significantly involved in the reduction in complex III. The last complex described is involved in the production of O_2_^-^ in the mitochondrial intermembrane space [56].

However, the production of ROS by the complexes may vary from organ to organ or even in pathological conditions [57,58,59]. Complexes I and III, for example, are the main generators of ROS in mitochondria, but complex III is the primary source of ROS production in the heart and lungs. In neurodegenerative diseases and aging, complex I is the primary source of ROS [60]. Under normal conditions, the activity of complex III is twice that of the first complex in generating ROS [56,61].

Typically, the production of oxygen radicals is a consequence of hyperoxia in tissues, which can lead to neurotoxicity in the brain due to their chemical reactivity with biological molecules. This reactivity results in changes in cellular function and cell death, processes that are involved in ROS imbalances [62]. Oxidative stress is a condition in which the balance between ROS and cellular antioxidant levels is disturbed, resulting in cellular damage due to the reactivity of these compounds with proteins, lipids, and nucleic acids [61,63,64,65]. To achieve this, highly specialized cells with high oxygen consumption, such as neurons and astrocytes, require an antioxidant system. In this case, antioxidant enzymes and non-enzymatic antioxidants control this balance. Superoxide dismutase (SODs) participate in the elimination of superoxide anion radicals, primarily derived from exogenous sources such as radiation, but also play a role in their production in the electron transport chain [66]. The family of isoenzymes called Glutathione peroxidases catalyzes the reduction in H_2_O_2_ or organic hydroperoxides to water or corresponding alcohols using reduced Glutathione (GSH) as an electron donor. Catalase, found in peroxisomes and mitochondria, is an enzyme that catalyzes the conversion of hydrogen peroxide into water [67,68].

Furthermore, reduced glutathione (GSH) and vitamin E are the primary non-enzymatic antioxidants. GSH is most abundant in the central nervous system, acting directly on free radicals, including superoxides, hydroxyl radicals, nitric oxide, and carbon radicals. Vitamin E, although not yet fully understood, is a fat-soluble molecule that appears to neutralize the cytotoxicity of hydrogen peroxide and lipid peroxidation in nerve cell membranes [62].

## 5. Oxidative Stress (ROS Damage to Neurons, Aβ/Tau Accumulation)

ROS generated by mitochondria have been implicated in acute brain injuries, such as stroke, as well as in neurodegeneration processes [69,70,71]. Therefore, oxidative stress generated by excess ROS or a failure of the antioxidant system serves as a modulator of neurodegenerative diseases, particularly through mitochondrial protein damage and lipid peroxidation [72,73], as well as through alterations in mitochondrial and nuclear DNA that affect protein expression [74]. In AD, beta-amyloid peptide (Aβ) accumulates primarily in extracellular regions [75]. However, this deposition can also be found within mitochondria, consistent with dysfunction [76,77]. This accumulation intensifies oxidative stress, resulting in a significant redox imbalance in neurons. This Aβ-induced oxidative damage is associated with increased by-products generated through lipid peroxidation (such as 4-hydroxynonenal), oxidized proteins (such as carbonyls), and nucleic acid damage (such as 8-hydroxydeoxyguanosine and 8-hydroxyguanosine) [78,79].

In a similar scenario, recurrent episodes of oxidative stress that are not resolved by the antioxidant system can increase the production and deposition of this peptide, promote phosphorylation, and intensify tau protein deposition in cases of AD [56,80]. In AD patients, tau protein undergoes hyperphosphorylation and intraneuronal accumulation, leading to a loss of its affinity for microtubules and destabilization of the cytoskeleton. Furthermore, its accumulation is strongly associated with the induction of oxidative stress and the formation of neurofibrillary tangles (NFTs), which compromise neuronal function [81]. One process that reduces tau hyperphosphorylation is mitophagy, a therapeutic target for patients [82].

In PD patients, high levels of oxidized lipids, proteins, and DNA are observed in the substantia nigra, along with heavy-metal toxicity that contributes to local ROS production. Lipid peroxidation in this region, in turn, worsens trace element (Fe^2+^) levels, which in turn cause neuronal damage [78]. Regarding PD, it is known that the interruption of the mitophagic pathway and the identification of autophagosomes containing damaged mitochondria are indicators of disease onset [80,82]. Mitophagy impairment in PD has been shown to result from mutations in α-synuclein, which accumulate in mitochondrial membranes.

Furthermore, neurons expressing the protein in its mutant form also signal the mitophagic process by exposing fragmented mitochondria and by increasing cardiolipin levels in the outer mitochondrial membrane [83]. Finally, in addition to compromising mitophagy, pathogenic α-synuclein interacts directly with mitochondria, causing mitochondrial dysfunction by blocking protein import, preventing membrane depolarization, contributing to oxidative stress, and affecting the electron transport chain [84,85,86]. The role of oxidative stress in ALS patients is not yet fully understood. However, the consequences of this process are well known and play a key role in the development of neuronal degeneration, as discussed in the previous section. Nevertheless, products of lipid peroxidation and nucleic acid oxidation have been observed in patients with the disease, demonstrating that oxidative damage is involved in the pathophysiology of ALS [78,87]. In HD, although the main cause is the toxicity promoted by mutant HTT, the exacerbated production of ROS has contributed to neural degeneration and disease progression. In this case, lipid oxidation, protein carbonylation, and DNA damage are hallmarks of the disease, with DNA damage activating cellular repair mechanisms in an undesirable way due to oxidation. This activation has side effects, including instability and expansion of CAG repeats in the mutated huntingtin gene, a key factor in HD pathogenesis [78,88].

This disease is also associated with impaired mitophagy, meaning that the mutated huntingtin protein affects the binding of adaptor proteins, such as optineurin (OPTN), through the AIM2 and RIPK1 pathways, as well as CALCOCO2, SQSTM1, and NBR1, to LC3. OPTN deficiency is also a contributing factor in the development of AD [89,90]. By disrupting these bonds, fusion between damaged mitochondria and autophagosomes is impaired, thereby impairing the efficient elimination of these defective organelles [82].

In general, the presence of oxidative stress markers in diseases affecting the nervous system results from the accumulation of dysfunctional mitochondria. This accumulation suggests impaired mitophagy, which is responsible for the clearance of damaged mitochondria via the PINK1/Parkin pathway, enabling metabolic homeostasis at the organellar level. Moreover, blocking mitophagy increases ROS levels, which, in turn, activate the NLRP3 inflammasome [91,92]. This intracellular protein complex induces the production of pro-inflammatory cytokines [82,93].

It is also worth mentioning that the dysregulation of lysosomes, membranous organelles that contain acid hydrolases with digestive functions, directly affects mitochondrial autophagy, as they fuse with autophagosomes and endosomes to degrade defective organelles [94]. It is also known that the functional deficit of these recycling organelles is involved in the abnormal accumulation of α-synuclein in PD, the aggregation of dysfunctional mitochondria, and, consequently, the activation of the intrinsic apoptotic pathway [94,95]. Neuroinflammation is a process characterized by inflammatory responses within the central nervous system triggered by brain injury, spinal cord injury, aging, autoimmunity, or diseases such as diabetes and ischemic episodes. These responses are activated by the release of pro-inflammatory cytokines, chemokines, second messengers, or by the accumulation of ROS [96].

**Figure 2:**
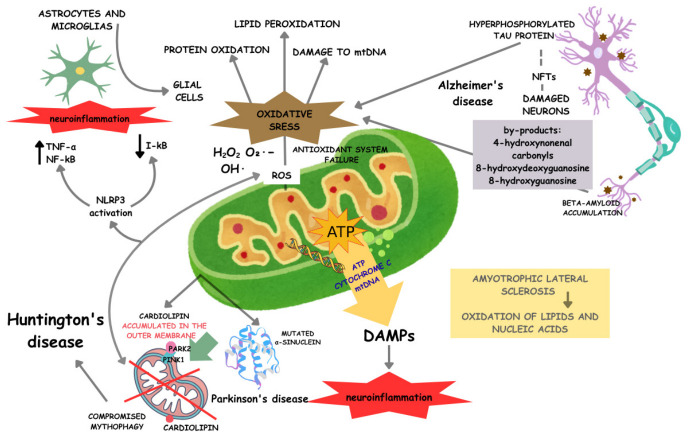
Under conditions of antioxidant deficiency, ROS accumulation leads to oxidative stress, resulting in protein and lipid oxidation and mitochondrial DNA damage. For each related neurodegenerative disease, some mechanisms increase oxidative stress. In PD, cardiolipin accumulates in the outer mitochondrial membrane, while α-synuclein undergoes mutation, compromising mitophagy. In HD, mutations in the huntingtin gene also compromise mitophagy, and mitochondrial trafficking is affected. This compromise, common to both diseases, leads to oxidative stress via increased ROS and to neuroinflammation via activation of the NLRP3 inflammasome. The increase in pro-inflammatory cytokines, particularly TNF-α, and the reduction in anti-inflammatory cytokines are detected by astrocytes and microglia, which then activate the inflammatory process. In addition, mitochondrial lysates such as ATP, mtDNA, and cytochrome c function as DAMPs, activating inflammatory responses. In AD, the accumulation of β-amyloid and tau proteins in their hyperphosphorylated forms promotes oxidative stress, leading to the formation of NFTs that impair neuronal function. Finally, in Amyotrophic Lateral Sclerosis, lipid peroxidation and oxidative damage to nucleic acids have been linked. Abbreviations: TNF-α, Tumor Necrosis Factor alpha; NF-κB, Nuclear Factor Kappa beta; I-κB, Inhibitor of kappa beta; NLRP3, NOD-like receptor family pyrin domain containing 3; ROS, Reactive Oxygen Species; ATP, Adenosine Triphosphate; DAMPs, Damage-Associated Molecular Patterns; NFTs, Neurofibrillary Tangles.

Among cytokines, the inflammatory mediator TNF-α stands out as a biomarker of neuroinflammation [97]. These signals are primarily captured by microglia and astrocytes, two types of glial cells that act as sensors and effectors of the inflammatory response. However, peripheral immune cells and endothelial cells are also involved in this process [98]. Thus, TNF-α and nuclear factor kappa B (NF-κB) protein levels increase and can trigger an inflammatory response in the nervous system. In contrast, I-κB protein levels decrease when nerve cells are exposed to mitochondrial lysates, indicating the progression of the inflammatory response [98]. This demonstrates that the release of mtDNA in cases of cellular injury or mitochondrial stress acts as a mitochondrial-derived damage-associated molecular pattern (DAMP), inducing inflammatory changes in microglia and, consequently, neuroinflammation [98] (Figure 2).

Mitochondrial dysfunction is a central feature of neurodegenerative diseases and should be understood as part of an integrated network involving altered energy metabolism, excessive reactive oxygen species (ROS) production, impaired mitophagy, and activation of neuroinflammatory pathways. Defective mitophagy, particularly via disruption of the PINK1/Parkin signaling axis, results in the accumulation of damaged mitochondria, enhancing oxidative stress and promoting the release of mitochondrial-derived danger signals. These events contribute to microglial activation and the upregulation of pro-inflammatory mediators through pathways such as NF-κB, thereby exacerbating neuronal injury and disease progression, as observed in AD and PD. In this context, physical exercise emerges as a key modulator of mitochondrial homeostasis, as it stimulates mitochondrial biogenesis via PGC-1α, improves mitophagic flux, enhances antioxidant defenses, and attenuates neuroinflammatory responses. Therefore, the interplay among mitochondrial dynamics, redox balance, and inflammation provides a mechanistic framework for explaining the neuroprotective effects of exercise [98,99,100,101,102,103].

## 6. The Role of Physical Exercise as a Mitochondrial Protector

Regular physical exercise is one of the non-pharmacological therapeutic interventions with a positive impact on physiological health and can be used to treat various metabolic disorders [99]. An active lifestyle can improve cognition and reduce the risk of neurodegenerative disorders [100,101]. These neuroprotective effects are absent in conditions such as dementia, AD, and PD [102,103]. Physical inactivity is recognized as a significant modifiable risk factor for the development of AD, as it contributes to mitochondrial dysfunction, increased oxidative stress, and impaired neuroprotective mechanisms [104]. At the cellular level, physical activity triggers processes such as mitochondrial biogenesis, reduced inflammation, neurogenesis, neuroplasticity, and increased oxygenation of brain tissue, which help combat cognitive decline in neurodegenerative diseases [105].

**Figure 3:**
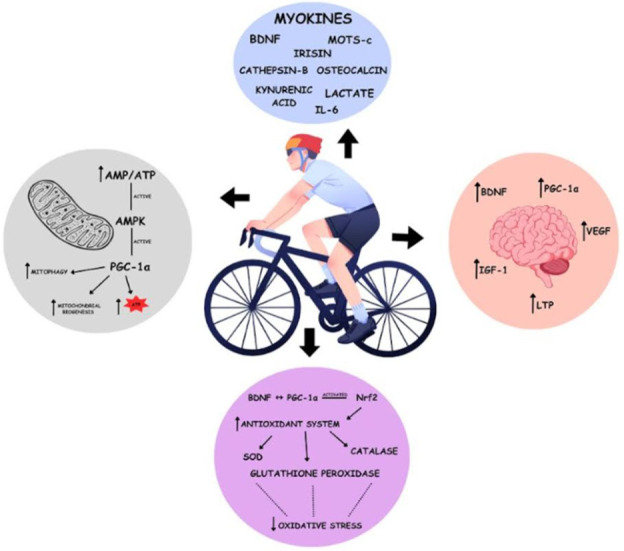
Physical exercise activates AMPK by increasing the AMP/ATP ratio, thereby controlling energy balance and mitophagy by stimulating ULK1. This activation stimulates PGC-1α, which regulates mitochondrial biogenesis, enhances antioxidant defense through activation of the Nrf2 pathway, and influences mitophagic processes. In addition, it releases substances that circulate in the bloodstream and can cross the blood–brain barrier, thereby contributing to cognitive improvement, neurogenesis, and synaptic plasticity. The increase in BDNF, in turn, is directly related to the activation of the PGC-1α pathway. Exercise also increases IGF-1 expression in brain regions associated with neurogenesis and hippocampal development. In response to exercise, VEGF contributes to cerebral angiogenesis. Abbreviations: BDNF, Brain-Derived Neurotrophic Factor; MOTS-c, Mitochondrial Open Reading Frame of the 12S rRNA-c; IL-6, Interleukin 6; AMP, Adenosine Monophosphate; ATP, Adenosine Triphosphate; AMPK, AMP-Activated Protein Kinase; PGC-1α, Peroxisome Proliferator-Activated Receptor Gamma Coactivator 1-Alpha; IGF-1, Insulin-Like Growth Factor 1; VEGF, Vascular Endothelial Growth Factor; Nrf2, Nuclear Factor Erythroid 2-Related Factor 2; SOD, Superoxide Dismutase.

In other words, exercise leads to mitochondrial adaptations that vary based on intensity, duration, and frequency. These changes encompass enhanced mitochondrial biogenesis, dynamic remodeling of mitochondrial networks through fusion and fission, cristae restructuring, mitophagy, and improvements in respiratory capacity and oxidative metabolism (Figure 3). This scenario leads to significant metabolic benefits, including elevated oxygen consumption, improved insulin sensitivity, and enhanced fatty acid oxidation. In addition to mitochondrial biogenesis, endurance exercise increases the area of mitochondrial inner membranes per mitochondrial volume in human muscles [106]. Therefore, it is essential to evaluate the effects of these activities, with a particular focus on mitochondrial function in the brain. Regular exercise plays a significant role in stimulating mitochondrial biogenesis by increasing adenosine monophosphate (AMP) levels, which activate AMPK. This enzyme acts as a sensor of energy status and coordinates energy production and expenditure in a balanced manner [107,108]. Subsequently, peroxisome proliferator-activated receptor gamma coactivator 1-alpha (PGC-1α), which also contributes to ROS control and antioxidant capacity, is stimulated by AMPK and regulates mitochondrial biogenesis [109]. On the other hand, the protein complexes of OxPhos adapt during physical activity, reflected in increased mitochondrial efficiency and energy production. These are complex 1 (NADH oxidoreductase) and complex IV (cytochrome c oxidase) [105,110,111].

This cellular mechanism primarily occurs in mitochondria associated with skeletal muscle, but its effects are similar in the brain. However, further studies are required to better understand these mechanisms [105]. It is worth noting that during physical exercise, ROS production tends to increase due to enhanced tissue oxygenation. However, at controlled levels, these species are positively involved in processes such as inflammation and apoptosis [105]. Exercise induces beneficial stress responses in mitochondria in a process known as mitohormesis. Exercise increases ROS production and triggers mitochondrial unfolded protein response (UPRmt). However, exercise-induced mitochondrial stress also stimulates the production and release of myomitokines such as growth differentiation factor 15 (GDF-15), and fibroblast growth factor 21 (FGF21), as well as mitochondrial DNA (mtDNA)-derived peptides (humanin and mitochondrial ORF of the 12S rRNA type-c (MOTS-c)), all of which have beneficial metabolic effects [112,113,114].

MOTS-c, produced and released by muscle during exercise, acts locally or in distant organs in a hormone-like manner. Although it is not yet known whether its intracellular and extracellular forms differ structurally, the peptide stimulates fatty acid oxidation and glucose uptake in skeletal muscle and adipocytes by activating AMPK. Its production is induced by exercise and is dependent on ROS, and MOTS-c can migrate to the nucleus, modulating gene expression related to stress adaptation, mitochondrial biogenesis, and dynamics [115,116,117].

Furthermore, oxidative stress is reduced through adaptations in endogenous mechanisms, including strengthening the antioxidant system to neutralize or reduce reactive species. The enzymes superoxide dismutase (SOD), glutathione peroxidase, and catalase exhibit enhanced activity. This modulation is influenced by the activation of the Nrf2 (nuclear factor erythroid 2-related factor 2) pathway by physical activity stimuli, increasing the expression of genes related to the antioxidant system [105]. Furthermore, evidence from animal models suggests that exercise-induced increases in BDNF levels are involved in synaptic plasticity and neuronal growth [118]. This neurotrophic factor, considered a type of myokine, has the potential to delay symptoms in neurodegenerative diseases such as those observed in AD and PD, as well as to enhance learning and memory [119]. Thoroughness related to the increase in this growth factor through physical activity and the highlighted benefits occurs by facilitating the induction of LTP through the activation of tropomyosin receptor kinase-B (TrkB) receptors in the postsynaptic region, especially in excitatory responses, and by acting in several signaling cascades involved in LTP. These cascades include activation of the Ras/extracellular signal-regulated kinase (ERK), phosphoinositide 3-kinase (PI3K)/Akt, and phospholipase C-γ (PLCγ)/inositol 1,4,5-triphosphate (IP3) pathways, which promote the phosphorylation of proteins such as CamKII and CREB, regulate the upregulation of BDNF, TrkB, and synapsin I, and modulate the function of NMDA and AMPA receptors, consolidating synapse and neuronal integrity [118]. It is also worth noting that BDNF directly regulates mitochondrial biogenesis by activating PGC1α, thereby increasing the availability of energy substrates for cells, such as ATP and NAD+, and improving synaptic function [120].

Exercise training induces beneficial adaptations across multiple organs, including adipose tissue. One of the most striking effects is the “beiging” or “browning” of inguinal subcutaneous white adipose tissue (iWAT) [121,122]. Under conditions of low thermogenic demand, adipocytes in this depot resemble classical white fat cells. However, stimuli such as cold exposure, a high-fat diet, or exercise promote their conversion into brown-like adipocytes, characterized by small, multilocular lipid droplets, elevated mitochondrial content and activity, enhanced glucose uptake, and increased expression of thermogenic genes, including PGC-1α, uncoupling protein-1 (UCP1), and PR domain containing 16 (PRDM16) [112]. These adaptations are less pronounced in visceral white adipose tissue and brown adipose tissue, indicating that exercise-driven thermogenesis mainly occurs in iWAT. Additionally, skeletal muscle exhibits non-shivering thermogenesis during exercise, likely mediated by sarcoplasmic reticulum calcium ATPase (SERCA) activity. This raises the intriguing question of why adipose tissue thermogenesis increases when muscle thermogenesis is already elevated [123,124].

On the other hand, it is known that PGC1α increases BDNF expression, as evidenced by decreased synapse formation and blocked synaptogenesis after PGC1α knockdown, revealing a mutually beneficial relationship [125].

It is worth noting that exerkins mediate the most-studied communication between skeletal muscles and the brain: secretory factors from any organ, in response to physical activity, that circulate through the bloodstream and can cross the blood–brain barrier (BBB) to influence mitochondrial dynamics and bioenergetics. Currently known exercins are released by skeletal muscle (myokines), liver (hepatokines), and adipose tissue (adipokines).

IL-6 and lactate are associated with skeletal muscle, increasing intracellular ATP levels and mitochondrial volume, and activating PGC1α [125]. Lactate is involved in memory processing and the formation of long-term memory [104]. The beneficial impact on memory and learning performance was also linked to increased hippocampal volume, attributed to elevated serum BDNF levels [126]. Irisin, expressed in the same organ, acts on energy expenditure, increases BDNF levels, and influences brain development [104].

Adiponectin, a type of adipokine, decreases ROS production and increases intracellular ATP levels. Finally, fibroblast growth factor 21 (FGF21) and β-hydroxybutyrate (BHB), a type of ketone body, are examples of hepatokines that also cross the BBB. The former decreases ROS production, increases respiration, and activates PGC1α. The latter increases the activity of complex II, activates PGC1α, and is involved in ROS production [125]. In summary, the myokines mentioned above, as well as cathepsin B, osteocalcin, and kynurenic acid, positively regulate BDNF expression, primarily in the hippocampus, and enhance cognitive function [104].

The role of exercise in the expression of the insulin-like growth factor-1 (IGF-1) gene in various brain regions was also analyzed. This growth factor is associated with neuronal formation and hippocampal development, which are involved in verbal communication and memory [126]. Along with other growth factors, such as vascular endothelial growth factor (VEGF), which crosses the BBB and contributes to cerebral angiogenesis in response to physical exercise, these factors mediate the benefits of physical activity in neurogenesis, memory, and the formation of new blood vessels [120].

Furthermore, evidence suggests that physical activity, particularly acute exercise, is directly associated with mitophagy. This occurs because the increased AMP/ATP ratio during exercise induces cellular energy stress, which, in turn, activates AMPK. This protein stimulates the ULK1 kinase, initiating the autophagic cascade [127]. At the same time, the mTORC1 complex, which inhibits mitochondrial autophagy, is suppressed, thereby favoring the elimination of dysfunctional organelles. Furthermore, markers such as LC3-II, p62, and ubiquitin are significantly increased after physical activity, which supports the evidence for the mentioned processes [127].

On the other hand, short-term exercise can contribute to the biogenesis of lysosomes and autophagosomes, which are crucial for mitophagy [94]. With chronic physical training, improvements in mitophagy flux are initially observed. However, this mechanism tends to decline with prolonged exercise adaptation, which improves mitochondrial quality [127].

Table 1 summarizes the types of exercise and their cognitive benefits and shows evidence that interventions with physical exercise, especially aerobic exercise, are effective in stabilizing cognitive decline and improving neurobiological markers in conditions such as mild cognitive impairment and AD. Figure 3 summarizes the process.

## 7. Clinical Evidence Supporting Exercise

A growing body of evidence from clinical trials and meta-analyses supports the role of physical exercise as a non-pharmacological intervention to mitigate cognitive decline and enhance brain health. In the systematic review and meta-analysis of 20 randomized clinical trials including older adults with mild cognitive impairment (MCI), intervention with multimodal and isolated exercises, including dance, walking, Tai Chi, and mind–body exercises, showed improvement in cognitive function according to increased MoCA scale scores, with moderate-intensity and long-term exercises (for more than 16 weeks) standing out in the cognitive improvement observed. Nevertheless, quantitative and statistical results confirm that physical exercise is a practical, non-pharmacological strategy for improving cognition in older adults aged 60 and older with mild cognitive impairment [133].

In a review and meta-analysis, the authors evaluated the impact of physical exercise interventions on cognitive and psychological outcomes in people with mild cognitive impairment (MCI). The authors combined data from several clinical trials and observed that exercise provides modest but consistent benefits for global cognitive function, memory, and depressive or anxiety symptoms in individuals with MCI. Strengths: The study aggregates evidence from multiple trials, which increases the statistical power and robustness of the conclusions. It addresses not only the cognitive aspect but also the psychological outcomes, enriching clinical interpretation. Additionally, it employs clear inclusion criteria and rigorous quantitative analysis. The bias risk for this study included trials that were heterogeneous in terms of type, intensity, and duration of interventions, which makes standardizing recommendations difficult; many studies involved small sample sizes, with a risk of bias; and the meta-analysis failed to address intrinsic limitations of the original studies (such as lack of blinding, protocol variability, and short follow-up) [134].

The studies below highlight the positive impact of various exercise regimes on cognitive function, neuroimaging biomarkers, and biochemical profiles in populations with or at risk for cognitive impairment. Some studies were summarized in Table 2 and Table 3.

The analysis demonstrates that physical activity (aerobic, resistance, and multimodal exercise) yields a significant positive effect on global cognition, evidenced by a standard mean difference. Subgroup analysis confirmed that aerobic exercise programs are consistently associated with moderate effects. Furthermore, sensitivity analysis revealed that the choice of the control group has a significant impact on the outcome. In conclusion, physical exercise, particularly aerobic training, benefits global cognition in MCI patients. However, evidence concerning specific cognitive domains and psychological measures remains insufficient, underscoring the necessity for future clinical trials with enhanced methodological rigor.

The studies included in Table 2 are summarized below.

**Table 2:** Clinical trials investigating the effects of exercise on cognitive function and biomarkers.

Type of Study	Duration	Intervention	Main Findings	Reference
Dementia Risk Study; *n* = 1235; Elderly	12 months	Multimodal exercise (aerobic + strength + balance).	Cognition: Improved global cognitive function (MMSE). Function: Enhanced ability to perform daily living activities.	[135]
Parkinson’s Study (SPARX); (*n* = 128, PD)	6 months	High-intensity treadmill training.	Cognition: Improved executive function and processing speed. Motor Function: Significant UPDRS-III improvement.	[136]
Meta-Analysis (MCI and Dementia)	-	Systematic review of 11 RCTs.	Physical exercise interventions consistently improve global cognitive function, with aerobic exercise showing the most robust effects.	[134]
Prospective, single-center, single-blind, randomized controlled trial, 48 patients with mild to moderate first ischemic stroke	3 months	Passive, active, resisted, and self-assisted range of motion exercises for joints (affected and unaffected sides); mobilization exercises including sitting up out of bed, standing, transferring, and walking	Reduction in inflammatory marker levels (IL-6, leukocytes, neutrophils, and monocytes); less motor impairment; improvement in cognitive performance	[137]
Secondary analysis of a randomized, controlled, multicenter clinical trial, *n* = 1679 individuals, 70 y or older with memory complaints but no diagnosis of dementia	3 years	Moderate-to-vigorous exercise.	Cognition: protective effects against neurodegenerative damage associated with p-tau181 protein accumulation in early interventions; reduced increase in p-tau181 concentrations over time.	[138]
Cross-sectional study with cognitively healthy individuals and individuals with cognitive impairment (*n* = 1144)	3 years	Vigorous and moderate activities. Walking	Reduction in plasma levels of p-tau217 and NfL; improvement in overall cognitive function; delay in cognitive decline associated with AD.	[139]
Randomized, double-blind clinical trial in 44 patients with coronary artery disease (CAD)	12 months	Moderate- and high-intensity aerobic exercises (HIIT, MICT)	Increase in BDNF levels; decrease in IGF-1; improvement in short-term and working memory	[140]
Secondary analysis of a randomized clinical trial involving 300 elderly individuals at risk of falling	6 months	Functional power training, balance and mobility training, and simultaneous cognitive activities	Reduction in β amyloid (1–40) levels; improvement in cognitive domains (reaction and choice, psychomotor skills, and attention)	[141]
Randomized controlled clinical trial (ENHANCE) with 88 cognitively healthy older adults living in urban and rural areas	12 months	Physical exercises aimed at muscle strength, balance, flexibility, and endurance.	Less reduction in gray matter volume (GMV) in regions above the left inferior temporal lobe; improvement in overall cognitive function (MoCA); induction of neuroplasticity	[142]
Randomized controlled clinical trial with adults after ischemic stroke; (*n* = 44)	3 months	Low- or moderate-intensity cycling training	Increase in VEGF-A; improvement in cognitive function (ACE-III)	[143]

MMSE, Mini-Mental State Examination; UPDRS, Unified Parkinson’s Disease Rating Scale; MCI, Mild Cognitive Impairment; MoCA, Montreal Cognitive Assessment; RCTs, Randomized Controlled Trials; IL-6, Interleukin-6; NfL, light chain neurofilaments; HIIT, High-Intensity Interval Training; DA, Alzheimer’s disease; MICT, moderate-intensity continuous training; ACE-III, Addenbrooke’s Cognitive Examination—III; BDNF, Brain-Derived Neurotrophic Factor; IGF-1, Insulin-like Growth Factor 1; VEGF-A, Vascular Endothelial Growth Factor A; CAD, coronary artery disease.

One randomized trial evaluated community-dwelling older adults with pre-frailty and frailty who underwent a multicomponent intervention (strength, endurance, balance, and coordination). The results showed partial reversal of frailty, improved cognition, and significant functional gains. The strengths include the practical, multidimensional intervention that accurately reflects real-world context, its positive impact on both physical and cognitive aspects, and its high clinical applicability in geriatrics. However, the trial was conducted at a single center with a moderate sample size, and adherence outside a supervised setting was not explored [135].

Some authors conducted a Phase 2 clinical trial to investigate the effect of high-intensity treadmill exercise on motor symptoms in patients newly diagnosed with PD. The results showed that intensive training was safe and well-tolerated and was associated with stabilization of motor symptoms compared with the control group. The strengths of this study include the innovative application of high-intensity exercise in a vulnerable population, promising findings in the early stages of the disease, and a well-controlled intervention. The bias does not impact non-motor outcomes (such as cognition or quality of life), and, as a Phase 2 trial, it requires replication in larger Phase 3 trials [136].

In another randomized controlled clinical trial, the potential benefits of moderate physical exercise on inflammatory modulation, initiated within the first 24 h after a mild to moderate ischemic stroke showed that patients undergoing ESV intervention had less marked elevations in proinflammatory markers, such as IL-6, leukocytes, neutrophils, and monocytes, on days 4 and 7 after stroke, compared to the usual care group (UCG). In addition, lymphocyte levels tended to increase in the VEE group, suggesting a more balanced inflammatory response. Fibrinogen showed a nonlinear pattern, with an initial increase followed by a more pronounced reduction in the VEE group, suggesting possible exercise-induced regulation of hemostasis [137].

In a secondary analysis of a randomized clinical trial, the relationships among blood p-tau181 levels, moderate-to-vigorous physical activity, and cognitive performance in older adults aged 70 years or older were examined over 3 years. The results showed that at the beginning of the study, there was no association between physical activity level and p-tau181 concentration. However, over time, more active individuals showed a slower increase in this biomarker, suggesting a possible cognitive protective effect against the accumulation of these proteins and their folding. On the other hand, the cognitive benefits of physical exercise ceased to be significant when the concentration of p-tau181 exceeded 9.36 pg/mL (cross-sectional analysis) or 3.5 pg/mL (longitudinal analysis) [138].

In a cross-sectional study in which cognitively healthy individuals and individuals with cognitive impairment were evaluated over a three-year period, research into moderate-to-vigorous physical activity, plasma biomarkers of AD, and cognition showed a significant reduction in levels of p-tau217 protein and neurofilament light chain (NfL) in the most active group compared to the lowest quartile (most sedentary group). The highest quartile still had higher Mini-Mental State Examination (MMSE) scores and lower Clinical Dementia Rating Scale (CDR-SB) scores, indicating improved cognitive function and a lower risk of dementia, respectively. It is worth noting that these relationships were more evident among participants aged 65 or older and those who already had some degree of cognitive impairment, compared to younger individuals without cognitive changes. There was no significant difference between the levels of glial fibrillary acidic protein (GFAP) and β-amyloid 42/40 (Aβ42/40) in both quartiles [139].

A randomized clinical trial investigated the effects of two periodized aerobic training protocols—linear (LP) and non-linear (NL)—in patients with stable coronary artery disease (CAD), focusing on cognitive functions and the neurotrophic biomarkers BDNF, IGF-1, and cathepsin-B. Over 36 supervised sessions, participants performed moderate- and high-intensity exercises, with assessments conducted before and after the intervention. Although neither protocol directly improved cognition, both significantly increased BDNF levels and reduced IGF-1, with no change in cathepsin-B. Furthermore, reduced IGF-1 was identified as an independent predictor of cognitive decline, suggesting that neurotrophic biomarkers may play a significant role in the cognitive response to physical exercise. The study highlights the importance of physical activity for patients with CAD, given the potential of targeted aerobic interventions to preserve cognition in these individuals [140].

A 6-month randomized clinical trial evaluated the effects of a dual-task power-based functional training program (DT-FPT) in older adults at increased risk of falls, living in independent communities. The protocol involved supervised group sessions twice a week, combining physical exercises for strength, balance, and mobility with simultaneous cognitive tasks. The main findings indicated significant improvements in reaction time and attention, as well as preservation of psychomotor skills, when compared to the control group that received usual care. However, no significant changes were observed in overall cognitive function, working memory, learning, or executive function. Regarding biomarkers, no consistent changes in inflammatory and neurological markers, except for a significant reduction in β-amyloid (1–40) levels. Furthermore, the cognitive and biological effects of the intervention were not influenced by BDNF-Met or ApoE-ɛ4 genotypes [141].

The ENHANCE study demonstrated that regular physical exercise, integrated with weekly multidomain sessions that include cognitive training and nutritional education, can confer significant benefits for cognitive function in older adults. Over 12 months, participants in the intervention group showed improvement in their Montreal Cognitive Assessment (MoCA) scores, indicating significant cognitive gains, especially among residents in rural areas. These effects were accompanied by evidence of neuroplasticity, with a smaller reduction in gray matter volume in the left inferior temporal lobe compared to the control group. In addition, geographic analyses revealed preservation of GMV in regions of the cerebellum and occipital cortex in the rural population, areas associated with motor coordination and visual processing, and in the left temporal-occipital fusiform cortex in the urban population, suggesting that physical exercise may act synergistically with cognitive stimuli to preserve brain circuits related to attention, memory, and processing speed [142].

The randomized controlled clinical trial investigated the effects of different intensities of aerobic training in adults during the post-ischemic stroke phase, comparing two groups undergoing ergometric cycling sessions: one at moderate intensity (MICT) and the other at light intensity (LICT), both combined with standard neurofunctional rehabilitation. In the cognitive domain, MICT participants demonstrated a significant increase in total ACE-III scores, particularly in attention and visuospatial skills. In addition, an increase in VEGF-A levels was observed, while peripheral BDNF levels decreased, possibly reflecting central redistribution [143].

Some studies support that exercise can interfere with mitochondrial function. The narrative review by Guzzoni et al. [144] was based on experimental models of rats selected for high (HRT) or low (LRT) response to physical training. The results demonstrate that aerobic exercise promotes significant mitochondrial adaptations in HRT animals, including increased citrate synthase activity, higher expression of PGC-1α, TFAM, NRF-1, and Lon protease, and improved antioxidant capacity and mitochondrial biogenesis. In contrast, LRT mice showed reduced mitochondrial activity after training, lower TFAM expression, increased Fis1, a marker of mitochondrial fragmentation, and higher production of ROS, indicating mitochondrial dysfunction and activation of pathways associated with mitophagy and apoptosis.

The studies included in Table 3 are summarized below. These studies were performed with humans and animals.

**Table 3:** Clinical Trials and Studies in Animal Models Supporting the Role of Exercise in Mitochondrial Function.

Population	Intervention	Duration	Main Findings	Reference
Clinical Trials				
44 healthy young and older adults	Endurance training (cycling, 4 times per week, moderate-high intensity)	12 weeks	Exercise increased mitochondrial protein expression and oxidative capacity, with a more pronounced effect in older adults	[145]
43 healthy young men	High-intensity interval training (HIIT) and moderate-intensity continuous training (MICT)	6 weeks	HIIT has a more pronounced effect on mitochondrial dynamics or respiratory function	[146]
Participants with type 2 diabetes mellitus	High-intensity interval training (HIIT)	12 weeks	HIIT increased acid sphingomyelinase activity, with baseline differences observed between men with type 2 diabetes and healthy controls. Exercise also modulated neutral sphingomyelinase expression, contributing to alterations in ceramide metabolism. These changes were associated with adaptations in mitochondrial quality control and remodeling in skeletal muscle.	[147]
385 healthy adults, obese individuals without other comorbidities	Aerobic endurance; High-intensity interval training (HIIT); combination of aerobic; sprint + strength	Analysis of acute responses; some studies ranged from 4 to 12 weeks	Increased expression of PGC-1α and mitochondrial biogenesis markers (TFAM, NRF-1/NRF-2/SIRT1, p53); increased respiratory capacity; and enzymatic activity of citrate synthase and complex I-IV enzymes	[148]
Animal models				
Preclinical study conducted with transgenic mice	Aerobic training on a treadmill	10 weeks	Decreased ROS production when exercise was associated with PGC-1α overexpression	[149]
Preclinical experimental study with female mice of the C57BL/6J strain	Progressive Weighted Wheel Running (PoWeR) + endurance	8 weeks of intervention with PoWeR + 3 days on the short-term protocol	Increased mitochondrial respiration rate in muscles: vastus lateralis, gastrocnemius, and triceps brachii; decreased mitochondrial sensitivity to ADP in muscles: gastrocnemius and vastus lateralis; increased H_2_O_2_ emission and decreased fractional electron leakage (FEL) in vastus lateralis; increased OxPhos in triceps	[150]
Study with male mice (C57BL/6J strain) and in vitro experiments with C_2_C_12_ muscle cells	Aerobic training on a treadmill	2 weeks	Increased expression of PGC-1α; improvement in mitochondrial function identified by mitochondrial respiration tests (OCR); Increased expression of proteins involved in OxPhos; complexes I (NDUFB8), II (SDHB), III (UQCRC2), and V (ATP5A)	[151]
Hypertensive mice (sample not specified in the study)	Aerobic training on a treadmill	8 weeks	Reduction in oxidative stress; increase in mitochondrial membrane potential; reduction in levels of Nox4 (superoxide-generating enzyme) and cytochrome c (marker of mitochondrial apoptosis)	[152]
Male Sprague-Dawley (SD) rats after induction of cerebral ischemia	Aerobic training on a treadmill	14 days	Increased expression of SIRT3 protein; Increase in PGC-1α, NRF-1, TFAM, Mnf2; Decrease in Drp	[153]

PGC-1α, Peroxisome proliferator-activated receptor gamma coactivator 1-alpha; TFAM, Mitochondrial transcription factor A; NRF-1, Nuclear respiratory factor 1; NRF-2, Nuclear respiratory factor 1; SIRT1, Sirtuin 1; p53, Tumor protein p53; AMPK, AMP-activated protein kinase; ADP, Adenosine diphosphate; Nox4, NADPH oxidase 4; SIRT3, Sirtuin 3; ROS, Reactive Oxygen Species; OxPhos, Oxidative Phosphorylation; H_2_O_2_, hydrogen peroxide.

In a randomized clinical trial, young and elderly individuals undergoing different types of physical training were evaluated, demonstrating that both aerobic and interval exercise increased mitochondrial protein translation and improve metabolic adaptations, especially in older adults. Strengths: The use of advanced proteomic techniques, comparisons between age groups, and relevant translational findings. Weaknesses: relatively small sample size, short-term intervention (12 weeks), and lack of follow-up to verify maintenance of effects [145].

The six-week training regimen induced a remodeling of the skeletal muscle mitochondrial network. The pre-training sparse and punctate network was transformed by MICT (moderate-intensity continuous training) into a grid-like structure with intermediate longitudinal connections. In contrast, HIIT resulted in a less distinct grid but a more strongly longitudinal network. Training also upregulated mRNA expression of mitochondrial fusion proteins and downregulated fission proteins, effects that were more marked following high-intensity interval training (HIIT). PGC-1α mRNA expression followed a similar trend, with variation between the one-day and six-week time points. Thus, HIIT and MICT elicit distinct patterns of mitochondrial adaptation, reflected in divergent network remodeling and molecular regulation. The results are interesting; however, they should be interpreted with caution due to the limited sample size [146].

In another study, the authors aimed to evaluate the effects of supervised effective exercise on skeletal muscle Acid sphingomyelinase, neutral sphingomyelinase, sphingolipids, and their association with mitochondria in humans with different degrees of insulin resistance and glucose tolerance. However, the study has biases, such as a relatively small sample size and a homogeneous participant demographic, restricted to overweight, middle-aged Caucasian men [147].

A preclinical study in adult Sprague-Dawley rats examined the effects of physical training on mitochondrial function and neurological recovery after cerebral ischemia. The animals underwent transient occlusion of the middle cerebral artery and were divided into groups with or without exercise intervention, with or without SIRT3 overexpression, or with SIRT3 silencing. The results at the mitochondrial level suggest that physical exercise positively regulates SIRT3 expression, thereby improving mitochondrial repair and quality control (MQC) by increasing mRNA levels of PGC-1α, NRF-1, and TFAM. In addition, mitophagy was indirectly enhanced by physical training through increased expression of PINK1 and Parkin proteins, as well as a better balance between mitochondrial fission and fusion. In this case, the expression of Drp1 and Fis, mitochondrial fission proteins, decreased, while that of OPA1 increased. These findings reinforce the effectiveness of physical training in regulating mitochondrial function via SIRT3 [153].

Another preclinical experiment was conducted in mice with skeletal muscle-specific PGC-1α overexpression. The objective was to analyze the influence of this overexpression, both with and without physical training, on mitochondrial function and intestinal microbiome (colon) composition. The animals were divided into four groups (control and exercise, with and without PGC-1α overexpression) and subjected to treadmill aerobic training. At the mitochondrial level, the results showed that the combination of exercise and PGC-1α overexpression significantly reduced basal ROS production induced by succinate in colon mitochondria, particularly hydrogen peroxide. In addition, the same combination improved the dynamics of the animals’ intestinal microbiome [149].

The preclinical study conducted in female C57BL/6J mice evaluated the effects of PoWeR (Progressive Weighted Wheel Running) exercise on skeletal muscle and mitochondrial adaptations. The animals underwent voluntary wheel-running training with progressive overload. At the mitochondrial level, the results showed higher rates of mitochondrial respiration in the vastus lateralis, triceps brachii, and gastrocnemius muscles, with the highest frequency in the former. In addition, both the gastrocnemius and vastus lateralis muscles showed lower mitochondrial sensitivity to ADP, indicating lower mitochondrial reactivity to small fluctuations in ADP. Also in the vastus lateralis, there was greater mitochondrial hydrogen peroxide emission, but lower fractional electron leakage (FEL), indicating greater mitochondrial efficiency. For the other muscles, the difference in FEL was not significant. Finally, an increase in OxPhos subunits was identified in the gastrocnemius, quadriceps, plantar, and triceps muscles, with a notable increase in OxPhos in the triceps [150].

A preclinical study examined the effects of aerobic training on skeletal muscle mitochondrial function, particularly the enzyme Aldehyde Oxidase 1 (AOX1). After two weeks of treadmill training, the results demonstrated a significant improvement in mitochondrial function in the animals, identified by increased respiration, increased expression of PGC-1α, and proteins related to OxPhos, including complexes I (NDUFB8), II (SDHB), III (UQCRC2), and V (ATP5A). Furthermore, the study also identified an increase in the proportion of oxidative fibers, which are rich in mitochondria and essential for long-duration activities [151].

Another preclinical study examined the effects of physical exercise on mitochondrial function in hypertensive conditions, using exosomes derived from endothelial progenitor cells (EPCs). The objective of the study was to understand the impact of an exercise intervention on the compositional and functional modifications of exosomes, with an emphasis on neuronal protection, in an in vitro model of angiotensin II- and hypoxia-induced injury. At the mitochondrial level, exosomes from hypertensive mice subjected to eight weeks of treadmill exercise (R-EPC-EXET) showed improved incorporation by N2a neuronal cells and restoration of miR-27 levels, a modulator of mitochondrial function. The increase in miR-27a reduced ROS production, improved mitochondrial membrane potential in damaged N2a cells, and decreased levels of cytochrome c and Nox4, markers of apoptosis [152].

A systematic review with meta-analysis investigated the impacts of physical exercise on mitochondrial dynamics, emphasizing mitochondrial biogenesis in skeletal muscle associated with PGC-1α expression. Thirteen randomized clinical trials involving healthy adults with different types, intensities, and durations of exercise were included in the study. The results demonstrated increased PGC-1α expression after resistance exercise. Interval and continuous training showed no significant differences in marker expression, and both induced mitochondrial biogenesis. In addition, other markers were associated, including TFAM, NRF-1, and NRF-2, as well as improved protein synthesis and performance of the respiratory chain, especially after high-intensity training. The study reinforces the need for further research to improve training strategies associated with mitochondrial responses [148].

Table 2 and Table 3 present clinical evidence demonstrating the beneficial role of physical exercise in cognitive function. Trials suggest that structured interventions, particularly aerobic exercise, can stabilize or mitigate cognitive decline in individuals with MCI and AD. These benefits are corroborated by biological and neuroimaging markers, including reduced cerebrospinal fluid tau pathology and slowed hippocampal atrophy. Similarly, studies in other conditions, such as PD, reveal improvements not only in motor but also in cognitive function. These results demonstrate that exercise serves as an adjunctive strategy for preserving brain health, underscoring its importance in management protocols for neurodegenerative diseases.

## 8. Conclusions

Mitochondrial dysfunction represents an important contributor to cognitive decline, interacting with other key mechanisms such as neuroinflammation, synaptic dysfunction, and metabolic alterations. Alterations in mitochondrial dynamics, impaired bioenergetics, increased oxidative stress, and defective mitophagy contribute to neuronal vulnerability and functional impairment. In this context, physical exercise emerges as a promising non-pharmacological strategy that modulates several pathways, including mitochondrial biogenesis, antioxidant defenses, and metabolic regulation. However, the relationship between mitochondrial adaptations and cognitive outcomes remains complex and not fully elucidated, particularly in human populations. Therefore, while current evidence supports a beneficial role of exercise in maintaining brain health, further studies are needed to clarify causal mechanisms and optimize intervention strategies.

## 9. Future Directions

There are several challenges associated with the role of mitochondria and physical exercise in preventing cognitive decline. Future research could involve converting the robust evidence of exercise-induced mitochondrial protection into personalized therapeutic strategies. A critical and immediate next step is the establishment of large-scale, multicenter, double-blind, randomized clinical trials to rigorously quantify the efficacy of exercise interventions across different stages of cognitive impairment and neurodegenerative diseases. Beyond exercise alone, future research should focus on synergistic approaches. This includes the development of standardized nutraceuticals and phytopharmaceuticals targeting mitochondrial pathways, such as compounds that activate AMPK or PGC-1α, for use as adjuvants to physical activity.

Furthermore, exploring the integration of these lifestyle and nutraceutical interventions with conventional pharmacological therapies—for instance, as an adjuvant to anti-CGRP antibodies in migraine-related cognitive fog or to optimize metabolic health in patients using triptans—represents a promising frontier for holistic patient management. Ultimately, the goal is to leverage multi-omics approaches to create personalized profiles, enabling the prescription of precise “exercise cocktails” combined with targeted supplements and conventional drugs. This paradigm shift, supported by rigorous evidence, will transform the management of cognitive decline from a reactive model into a proactive and precision-based model.

## Figures and Tables

**Table 1:** Different Types of Exercise and Their Mitochondrial and Cognitive Benefits.

Exercise Type/ Frequency	Mitochondrial Effects	Cognitive Impact	References
Short-term exercise		Increased lysosomal and autophagosome biogenesis	[94]
Long-term exercise (treadmill)	Decreased ROS levels	Increased antioxidants such as superoxide dismutase 1 and Glutathione peroxidase in the hippocampus, BDNF regulation via the ROS pathway	[102]
Voluntary exercise	Increased oxygen consumption and ATP production through the respiratory chain	Increased neurogenesis, Enhancement of dopaminergic effects in the substantia nigra in patients with PD	[128]
Short intense exercise (sprint)	Activation of anaerobic and aerobic pathways in ATP Production, Increased acetyl-CoA production and pyruvate dehydrogenase (PDH) activation	Improved cognition, antioxidant defense, and reduced neuroinflammation	[105]
Aerobic endurance exercise below 100% of VO2 max	Increased mitochondrial Ca^2+^ concentration with consequent activation of Krebs cycle enzymes, Reduction in oxidative stress	Reduced neuroinflammation by reducing pro-inflammatory cytokines, Increased brain health	[105]
Daily swimming training for 90 min over 6 weeks	Increased mtDNA, improved complex I and IV activities, and improved mitochondrial fission by increasing Drp1	Increased mitophagy via Parkin, Increased antioxidants (SOD-1), Increased PGC-1α	[129]
High-intensity training integrated with boxing, with 30 min of exercise and 15 min of boxing		Exercise prevented the expected decrease in dopamine transporters (DAT) in both the substantia nigra and putamen in PD patients, Exercise contributed to increased neuromelanin in the substantia nigra in the same patients	[130]
Exercises on exercise bikes/cross trainers/treadmills		Decreased inflammatory mediators, such as IL-6, in patients with AD	[129]
Moderate aerobic exercise for 40 min 3 times a week		Increased hippocampal volume, improved memory formation, and increased BDNF in healthy individuals	[131]
HIIT 5 days/week for 6 weeks		Increased hippocampal neurogenesis and decreased pro-inflammatory cytokines such as GM-CSF, IL-1β, and IL-7	[132]

ROS, Reactive Oxygen Species; BDNF, Brain-Derived Neurotrophic Factor; PD, Parkinson’s Disease; mtDNA, mitochondrial DNA; Drp1, Dynamin-related protein 1; SOD-1, Superoxide dismutase 1; PGC-1α, Peroxisome proliferator-activated receptor gamma coactivator 1-alpha; IL-6, Interleukin-6; AD, Alzheimer’s disease; HIIT, High-Intensity Interval Training; GM-CSF, Granulocyte–macrophage colony-stimulating factor; IL-1β, Interleukin-1 beta; IL-7, Interleukin-7.

## Data Availability

No new data were created or analyzed in this study. Data sharing is not applicable to this article.
